# OX40 Agonists and Combination Immunotherapy: Putting the Pedal to the Metal

**DOI:** 10.3389/fonc.2015.00034

**Published:** 2015-02-16

**Authors:** Stefanie N. Linch, Michael J. McNamara, William L. Redmond

**Affiliations:** ^1^Robert W. Franz Cancer Research Center, Earle A. Chiles Research Institute, Providence Portland Medical Center, Portland, OR, USA

**Keywords:** OX40, CTLA-4, PD-1, co-stimulation, immunotherapy, cancer

## Abstract

Recent studies have highlighted the therapeutic efficacy of immunotherapy, a class of cancer treatments that utilize the patient’s own immune system to destroy cancerous cells. Within a tumor the presence of a family of negative regulatory molecules, collectively known as “checkpoint inhibitors,” can inhibit T cell function to suppress anti-tumor immunity. Checkpoint inhibitors, such as CTLA-4 and PD-1, attenuate T cell proliferation and cytokine production. Targeted blockade of CTLA-4 or PD-1 with antagonist monoclonal antibodies (mAbs) releases the “brakes” on T cells to boost anti-tumor immunity. Generating optimal “killer” CD8 T cell responses also requires T cell receptor activation plus co-stimulation, which can be provided through ligation of tumor necrosis factor receptor family members, including OX40 (CD134) and 4-1BB (CD137). OX40 is of particular interest as treatment with an activating (agonist) anti-OX40 mAb augments T cell differentiation and cytolytic function leading to enhanced anti-tumor immunity against a variety of tumors. When used as single agents, these drugs can induce potent clinical and immunologic responses in patients with metastatic disease. However, each of these agents only benefits a subset of patients, highlighting the critical need for more effective combinatorial therapeutic strategies. In this review, we will discuss our current understanding of the cellular and molecular mechanisms by which OX40 agonists synergize with checkpoint inhibitor blockade to augment T cell-mediated anti-tumor immunity and the potential opportunities for clinical translation of combinatorial immunotherapeutic strategies.

## Introduction

Immunotherapy has become a major focus of anti-cancer therapy regimens and for good reason: when it works, patients can have long-lasting anti-tumor immune responses that not only eradicate primary tumors but metastatic lesions as well. Recently, investigators have focused on harnessing the power of checkpoint inhibitors, such as CTLA-4 and PD-1, to release the “brakes” on an anti-tumor T cell response through antibody-mediated antagonism of these receptors. However, checkpoint inhibition alone is not sufficient to promote tumor regression in a majority of patients. Generating a robust therapeutic immune response requires not only releasing the “brakes” but also stepping on the “gas.” T cell co-stimulation through receptors, like OX40 or 4-1BB, provides a potent “go” signal that actively promotes the expansion and proliferation of killer CD8 and helper CD4 T cells. Here, we discuss recent advances in the field of OX40 immunotherapy and the promise of triple combination therapy in the (not so distant) future.

## OX40: Stepping on the Gas

OX40 (CD134; TNFRSF4) is a member of the TNFR super-family and was originally characterized as a receptor that was primarily expressed by rat CD4 T cells from the thymus and lymph nodes following stimulation with concanavalin A (1). Subsequent research demonstrated that in both mice and humans, OX40 is expressed by CD4 and CD8 T cells during antigen-specific priming (2–5). OX40 expression is induced following TCR/CD3 cross-linking, and by the presence of inflammatory cytokines, including IL-1, IL-2, and TNF-α. The expression of OX40 following antigen encounter is largely transient for both CD4 and CD8 T cells (24–72 h), with the duration of OX40 expression by CD8 T cells reported to be shorter than for CD4 T cells (6). In the absence of activating signals, relatively few mature T cell subsets have been shown to express OX40 at biologically relevant levels (7). However, the constitutive expression of OX40 by follicular helper CD4 T cells (Tfh) has been described in both mice and humans (8–11). Within germinal centers, the CD4^+^/CXCR5^+^/CCR7^−^subpopulation of Tfh cells have been shown to have the highest level of OX40 expression and are thought to be important regulators of antibody production (12–14). In mice, OX40 is also constitutively expressed on FoxP3^+^ regulatory T cells (Treg cells), in contrast to human Treg cells where its expression is inducible (7). In contrast, antigen-specific activation can induce OX40 expression by numerous subsets of differentiated CD4 and CD8 T cells. In a murine model system (OT-II), Th1 and Th17 cells were both capable of a similarly robust induction of OX40 in response to peptide-activation (15). In humans, a substantial proportion of tumor-infiltrating CD4 T cells express OX40, presumably due to recognition of tumor antigens, and the frequency of OX40^+^ CD4 T cells may be prognostic for patient outcomes (16, 17). Similarly, activated peripheral CD8 T cells have also been shown to express OX40 in mice and humans (18, 19).

Ligation of OX40 on CD8 and conventional (non-regulatory) CD4 T cells, using either its natural ligand (OX40L) or agonist antibodies, promotes their survival and expansion. Evidence of this comes from studies using OX40- and OX40L-deficient mice, which are discussed in detail in several recent reviews (7, 20). These studies demonstrated that OX40- or OX40L-knockout mice had reduced expansion of both CD4 and CD8 T cells, combined with defective memory responses following antigen challenge, indicating the importance of endogenous OX40 expression in regulating T cell expansion (20–25). Furthermore, treatment with agonist anti-OX40 monoclonal antibodies (mAbs) along with TCR stimulation in wild-type animals induced expansion, differentiation, and increased survival of CD4 and CD8 T cells. Likewise, depletion of CD8 or CD4 T cells eliminated the ability of anti-OX40 mAbs to induce tumor regression in several tumor models (23, 24, 26–28). One study demonstrated that anti-OX40 administration was sufficient to overcome CD8 T cell tolerance to a self-antigen and restored their cytotoxic activity, highlighting the therapeutic potential for OX40 agonists (29). This is of particular importance for patients with cancer, as T cell tolerance to the tumor is a major obstacle for therapeutic modalities. Another group has demonstrated that enhanced CD8 T cell function following anti-OX40 treatment was mediated by the induction of CD40L expression on effector T cells thereby promoting DC maturation, because CD40^−/−^ mice have significantly fewer CD11c^+^ dendritic cells that migrate into the draining lymph nodes following anti-OX40 mAb (30). In fact, CD40^−/−^ mice treated with anti-OX40 mAbs all succumb to their tumors in contrast to wild-type mice, which have a 60% survival rate, suggesting the importance of CD40 expression following OX40 stimulation. Collectively, these data suggest that exogenous manipulation of OX40 signaling can boost stagnant T cell responses.

Several investigators have conducted studies to determine the mechanism by which OX40 promotes T cell survival. It has been demonstrated that following activation, OX40-deficient CD4 T cells failed to sustain expression of the anti-apoptotic proteins Bcl-x_L_ and Bcl-2. Moreover, the survival of activated CD4 T cells was rescued by retroviral transduction of Bcl-x_L_ or Bcl-2 (23). Sustained expression of Bcl-x_L_ was also necessary for the survival of tumor-reactive CD8 T cells following OX40 co-stimulation (31). Subsequent studies demonstrated that OX40 signaling in T cells induced expression of Survivin, and this was required to regulate and sustain T cell division over time. Survivin expression was maintained via the sustained activation of PI3K and PKB by OX40 signaling (32). However, Survivin expression does not supersede the requirement for Bcl-x_L_ and Bcl-2 following OX40 signaling in order to inhibit T cell apoptosis. Enhanced expression of Survivin and Bcl-2 family members is mediated via activation of IκB kinase and NF-κB1 following OX40 signaling (33). Other investigators have shown that TRAF2 is required following OX40 signaling in antigen-specific CD4 T cells, as the expression of a dominant negative TRAF2 in CD4 T cells inhibited their expansion, survival, and cytokine production (34). One of the functions of TRAF2 appears to be to prevent CTLA-4 expression following T cell co-stimulation through OX40, as CTLA-4 blockade at the time of T cell priming with antigen and anti-OX40 mAbs partially restored defective expansion in mice expressing a dominant negative TRAF2 protein. It remains unknown whether the same TRAF adaptors and NF-κB pathways are activated in T cells following ligand binding by other TNFR family members, such as CD27 and GITR (35, 36). Similarities and differences in the signaling pathways activated by T cell co-stimulatory receptors, including both TNFR family members, like OX40 and CD27, and immunoglobulin super-family members, like CD28 and B7 families, has been reviewed extensively elsewhere (37). The activation of multiple pathways by both co-stimulatory receptor super-families results in enhanced cell growth and effector function, and improves survival (37). Numerous investigators are currently testing the modulation of these receptors for various clinical applications and immunotherapies.

Preclinical studies demonstrated that treatment of tumor-bearing hosts with OX40 agonists, including both anti-OX40 mAb and OX40L-Fc fusion proteins, resulted in tumor regression in several preclinical models (20, 26, 27, 38–40). Recent studies have investigated the mechanisms by which these agonists function. In addition to promoting effector T cell expansion, since OX40 is constitutively expressed on Treg cells, OX40 agonists have the ability to directly regulate Treg cells. There are conflicting reports on whether these agonists promote or diminish Treg cell responses. Some have observed that anti-OX40 mAbs blocked the suppressive function of Treg cells *in vivo*, while others have observed Treg cell expansion (27, 41–43). These studies suggest that anti-OX40 can push Treg cells in both directions, depending upon the context of stimulation and the cytokine milieu. Indeed, the importance of the OX40 co-stimulatory pathway in regulating immunity is exemplified by the presence of autoimmune-like disease in mice with constitutive expression of OX40L (44, 45).

OX40 signaling has also been shown to inhibit the production of IL-10 by and suppressive function of Treg cells (46). Supporting these data, administration of anti-OX40 mAbs prior to tumor engraftment rendered Treg cells functionally inactive through inhibition of IL-10 production and elimination of Treg cell-mediated suppression of CD8 T cell responses (27, 30, 41). One recent report observed that cells expressing activating FcγR were required for the selective depletion of Treg cells from tumors, while there was no change in Treg cells in the draining lymph nodes at day 5 following anti-OX40 therapy (47). Other studies confirm that even at later time points following anti-OX40 treatment, there is no change in the frequency of Treg cells in the draining lymph nodes, so this effect may be localized to the tumor (27). In fact, this effect may be transient, as another report showed that at day 7 there was no difference in Treg cell frequency in the tumor between control-treated and anti-OX40-treated mice using the same CT26 colon cancer model (28). This study in particular also suggests that the immunological effects of anti-OX40 therapy can vary based on the tumor model examined; thus, one must be cautious of making generalizations regarding the precise mechanism of OX40 agonists. Other studies report that anti-OX40 mAbs reduce the suppressive activity of Treg cells *in vitro* and *in vivo* (27, 41). Whether anti-OX40 functions via Treg cell suppression, deletion, or both, treatment with these agonists should diminish the inhibitory effects mediated by Treg cells and thereby promote anti-tumor CD8 T cell responses necessary to maintain long-term anti-tumor immune responses. It is likely that multiple mechanisms are important for the anti-tumor activity of OX40 agonists.

**Figure 1:**
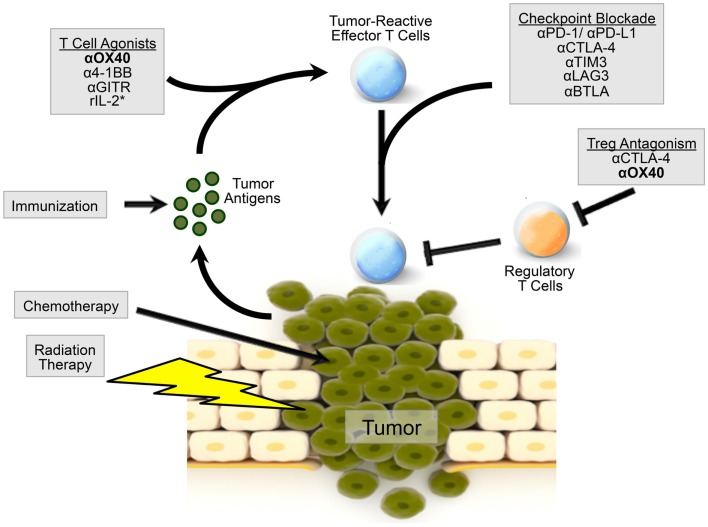
**Model of OX40 agonism in combination immunotherapy radiation and chemotherapy can induce the release of tumor-associated antigens by the tumor**. Patients can be immunized against these tumor-associated antigens to induce a robust immune response. Agonism of OX40, as well as other co-stimulatory molecules, can boost the generation of tumor-reactive effector T cells. OX40 agonism combined with checkpoint inhibition, via CTLA-4 or PD-1 blockade, or additional immunotherapy can further augment an effector T cell response. OX40 agonism can also inhibit Treg cell function, alleviating immunosuppression in the tumor microenvironment. OX40 agonism with combination therapy may provide a microenvironment more amenable to direct killing by effector T cells. *r denotes recombinant IL-2.

The ability of OX40 agonists to regulate immune responses, as well as the expression of OX40 on CD4 and CD8 lymphocytes from the tumors and tumor-draining lymph nodes in mice and humans (38, 40, 48), led investigators to examine OX40 manipulation as a treatment for cancer patients. Recently, the use of anti-OX40 monotherapy was tested in a Phase 1 trial in patients with solid tumors, with promising results (49). Twelve out of 30 patients receiving an OX40 agonist had regression of at least 1 metastatic lesion with only 1 cycle of treatment. Patient toxicities were much milder for anti-OX40 mAbs compared to more severe toxicities, i.e., autoimmune-like disease, colitis, etc., caused by treatment with CTLA-4 blockade (ipilimumab), and most frequently included a temporary lymphopenia. Patients receiving the OX40 agonist had an expansion of CD4 (non-Treg cells) and CD8 T cells following drug infusion with concomitant expression of activation markers CD38 and HLA-DR. Unlike treatment with ipilimumab, treatment with an OX40 agonist did not induce expansion of Treg cells either in the blood or the tumor (49, 50). What investigators did observe was that two out of three patients had IFN-γ-producing CD8 T cells following stimulation with autologous tumor cell lines *in vitro*, suggesting a tumor-specific T cell response, though the antigens they recognize remain unknown. Unfortunately, the development of human anti-mouse antibodies to the drug precluded continued treatment (49). MedImmune has several Phase 1 clinical trials investigating OX40 agonists including NCT02318394, NCT02205333, and NCT02221960. Indeed, the use of OX40 agonists in the clinic represents an exciting new chapter in cancer immunotherapy. Further studies and patient immune monitoring will provide further insight into the mechanisms by which OX40 agonists enhance an anti-tumor immune response. Nevertheless, despite all the positive data supporting the use of OX40 agonists in cancer, it is unlikely that anti-OX40 alone will be sufficient to cure all patients or all tumor types. However, there is great promise that combination immunotherapy incorporating both OX40 and checkpoint inhibition may be able to do what single agents alone cannot (Figure 1; Table 1).

**Table 1:** **Summary of animal studies using OX40 agonists alone or in combination**.

OX40 agonist	Combination	Cancer model	Curative	Key findings	Reference
OX86	a4-1BB	MethA sarcoma	Yes	Significant survival benefit; boosted T cell response	(51)
OX86	a4-1BB, adoptive cell therapy	B16-F10 melanoma	Yes	Significant survival benefit; monotherapy ineffective; boosted T cell response	(52)
OX86	a4-1BB, aPD-L1	c-Myc Tg hepatoma	Yes	Significant survival benefit; boosted T cell response	(53)
OX86	a4-1BB, immunization	N202.1A mammary	Yes	Significant survival benefit	(54)
OX86	aCD25	CT26 colon	No	Monotherapy alone effective; aCD25/aOX40 combo eliminated survival benefit of monotherapy	(27)
OX86	aCTLA-4	TRAMP-C1 prostate	Yes	Significant survival benefit; boosted T cell response; induced Th2 cytokine production by CD4	(55)
OX86	aCTLA-4	MCA-205 sarcoma	Yes	Significant survival benefit	(55)
OX86	aCTLA-4, aIL-4	TRAMP-C1 prostate	Yes	Significant survival benefit for triple combo; aOX40/aCTLA-4 combo also effective but less than triple	(55)
OX86	aCTLA-4, CpG	A20 lymphoma	Yes	Significant survival benefit for triple combo; monotherapy also effective but less than triple combo	(56)
OX86	aCTLA-4, CpG	4T1 mammary	Yes	Significant survival benefit; reduced metastases	(56)
OX86	Adoptive cell therapy	E.G7 thymoma	Yes	Significant survival benefit; monotherapy ineffective; boosted T cell response	(57)
OX86	Adoptive cell therapy	TRAMP-C1 prostate	No	Prolonged survival; monotherapy ineffective	(24)
OX86	Adoptive cell therapy, immunization	TRAMP-C1 prostate	No	Prolonged survival; monotherapy ineffective; boosted T cell response	(29)
OX86	aDR5, aCD40, a4-1BB (trimAb)	4T1 mammary	No	No effect of adding aOX40 to trimAb	(58)
OX86	aPD-1	ID8 ovarian	Yes	Significant survival benefit; monotherapy ineffective; boosted T cell response; reduced MDSCs	(59)
OX86	Arginase inhibitor	MCA-205 sarcoma	Yes	Significant survival benefit; monotherapy marginally effective; boosted T cell response; reduced MDSCs and TAMs	(60)
OX86	Caloric restriction	MCA-205 sarcoma	Yes	Significant survival benefit	(61)
OX86	CpG	TUBO mammary	No	Delayed tumor progression; prolonged survival	(62)
OX86	CpG and aCTLA-4, aGITR, or aFR4	A20 lymphoma	Yes	Significant survival benefit for aOX40/aFR4/CpG and aOX40/aCTLA-4/CpG combos; aOX40/aCTLA-4 combo ineffective; boosted T cell response	(63)
OX86	CpG, immunization	MOPC-21 myeloma	Yes	Significant survival benefit; monotherapy marginally effective; reduced IL-10 and Treg cells	(64)
OX86	Cyclophosphamide	B16 melanoma	Yes	Significant survival benefit; monotherapy ineffective; boosted T cell response	(65)
OX86	Cyclophosphamide, adoptive cell therapy	B16 melanoma	Yes	Significant survival benefit; aOX40/cyclophosphamide only marginally effective; boosted T cell response	(66)
OX86	Dasatinib	P815 mastocytoma	Yes	Significant survival benefit; monotherapy marginally effective; boosted T cell response	(67)
OX86	IL-12	MCA-205 H12 sarcoma	Yes	Significant survival benefit; monotherapy ineffective in aged mice	(68)
OX86	IL-12 transduced cells, immunization	A20 lymphoma	Yes	Significant survival benefit; monotherapy marginally effective	(69)
OX86	IL-12, a4-1BB	MCA26 colon	Yes	Significant survival benefit; monotherapy ineffective; boosted T cell response	(70)
OX86	IL-12, immunization	TRAMP-C1 prostate	Yes	Significant survival benefit	(68)
OX86	IL-2 complexes (rIL-2 + aIL-2)	MCA-205 sarcoma	Yes	Significant survival benefit; monotherapy marginally effective; prolongs survival and boosted T cell response in an anergy model	(71)
OX86	Immunization	NT2 mammary	Yes	Significant survival benefit; boosted T cell response	(72)
OX86	Immunization	B16-F10.9 melanoma	Yes	Significant survival benefit	(73)
OX86	None	B16-F10 melanoma	No	Significant survival benefit only with CD4 depletion; monotherapy ineffective	(74)
OX86	None	CT26 colon	Yes	Significant survival benefit; monotherapy ineffective in aged mice	(75)
OX86	None	MCA-205 H12 sarcoma	Yes	Significant survival benefit; monotherapy ineffective in aged mice	(75)
OX86	None	Renca renal	No	Prolonged survival	(76)
OX86	None	CT26 colon	No	Delayed tumor progression; prolonged survival	(76)
OX86	None	CT26 colon	Yes	Significant survival benefit when administered intra-tumorally; survival benefit eliminated in CD40^−/−^mice	(30)
OX86	None	CT26 colon	Yes	Significant survival benefit; reduced Treg cells via FcgammaR	(47)
OX86	None	CT26 colon	Yes	Significant survival benefit that requires CCR7	(27)
OX86	None	N2C mammary	Yes	Significant survival benefit	(27)
OX86	None	TSA mammary	Yes	Significant survival benefit	(27)
OX86	None	MCA-203 sarcoma	Yes	Significant survival benefit	(27)
OX86	None	BM185 leukemia	Yes	Significant survival benefit; boosted T cell response	(77)
OX86	None	A20 lymphoma	Yes	Delayed tumor progression	(63)
OX86	None	A31 lymphoma	No	Marginal effect on tumor progression	(78)
OX86	None	CT26 colon	No	Marginal effect on tumor progression	(78)
OX86	None	B16-F10 melanoma	Yes	Significant survival benefit	(79)
OX86	None	B16-D5 melanoma	No	No effect	(40)
OX86	None	MCA-205 sarcoma	Yes	Significant survival benefit	(40)
OX86	None	MCA-203 sarcoma	Yes	Significant survival benefit	(40)
OX86	None	GL261 glioma	Yes	Significant survival benefit	(40)
OX86	Radiotherapy	LLC lung	Yes	Significant survival benefit; monotherapy prolonged survival; boosted T cell response	(80)
OX86	Radiotherapy	3LL lung	Yes	Significant survival benefit	(26)
OX86	Radiotherapy, immunization	MCA-205 sarcoma	Yes	Significant survival benefit; monotherapy ineffective	(81)
OX86	Radiotherapy, immunization	GL261 glioma	Yes	Significant survival benefit; monotherapy ineffective	(81)
OX86	Surgical resection	MCA-205 H12 sarcoma	Yes	Significant survival benefit; boosted T cell response	(26)
Fc-OX40L	CpG, immunization	GL261 glioma	Yes	Significant survival benefit; boosted T cell response; survival dependent on B and NK cells	(82)
Fc-OX40L	CpG, immunization	GL261 glioma	Yes	Significant survival benefit; boosted T cell response	(83)
Fc-OX40L	None	MCA-205 sarcoma	Yes	Delayed tumor progression; boosted T cell response; induced OX40 expression on DCs	(84)
Fc-OX40L	None	B16-F10 melanoma	Yes	Significant survival benefit	(79)
Fc-OX40L	None	MCA-205 sarcoma	Yes	Significant survival benefit	(79)
Fc-OX40L	None	MCA-303 sarcoma	Yes	Significant survival benefit	(79)
Fc-OX40L	None	SM1 mammary	Yes	Significant survival benefit	(79)
Fc-OX40L	None	CT26 colon	Yes	Significant survival benefit	(39)
Fc-OX40L	None	RENCA renal	Yes	Significant survival benefit	(76)
Fc-OX40L	None	CT26 colon	Yes	Significant survival benefit	(76)
Fc-OX40L	Temozolomide, CpG, immunization	GL261 glioma	Yes	Significant survival benefit by adding temozolomide	(83)

## Combination OX40 Agonism and Checkpoint Blockade: Releasing the Brakes Only Goes so Far

### CTLA-4 blockade with OX40 agonism

In contrast to OX40, CTLA-4 is a negative regulatory surface molecule on T cells that competitively inhibits the CD28 co-stimulatory pathway by binding to B7-1 and B7-2. CTLA-4 is constitutively expressed on Treg cells and absent on naïve T cells, though expression is induced upon T cell activation as a means to attenuate and restrict T cell responses. This negative regulator is vital to prevent expansion of autoreactive T cells, as evidence by overt lymphoproliferative disease in CTLA-4 knockout mice (85, 86). Along these lines, inhibition of CTLA-4 using mAbs boosted effector CD4 and CD8 T cell function while inhibiting the suppressive function of Treg cells (87–91). These data led investigators to hypothesize that removing the “brakes” on a T cell response via CTLA-4 blockade would effectively allow the immune system to eliminate cancer cells and induce long-lasting anti-tumor immunity. Indeed, investigators have demonstrated the potency of checkpoint inhibition in cancer (90, 92–96). Clinical use of ipilimumab to block CTLA-4 has demonstrated improved survival in patients with metastatic melanoma (97–99). However, only a subset of patients treated with ipilimumab exhibit an objective clinical response (97). Thus, it is clear that additional strategies are necessary to improve patient outcomes and reduce lymphocyte dysfunction in cancer.

Investigators have tested whether combination immunotherapy, targeting both co-inhibitory and co-stimulatory molecules, is capable of overcoming tumor immune tolerance to induce a potent CD8 T cell response and ultimately tumor regression. The hypothesis behind this research is that because these molecules target distinct and also complementary pathways that tumor regression and the induction of a cytolytic T cell response may be amplified. Recent data from our laboratory indicated that combined anti-OX40/anti-CTLA-4 mAb therapy dramatically improved survival in the poorly immunogenic TRAMP-C1 prostate and the more immunogenic MCA-205 sarcoma models. Specifically, this combination therapy induced robust effector CD4 and CD8 T cell responses necessary to induce tumor regression (55). Likewise, Marabelle et al. recently demonstrated that combined anti-OX40/anti-CTLA-4 (with adjuvant CpG) was capable of inducing regression of local and distant tumors using several aggressive tumor models when administered intra-tumorally. The mechanism for intratumoral administration of combination therapy appears to be through depletion of Treg cells at the tumor site, allowing for a greater influx of CD8 T cells into the tumor (56). In contrast, when this combination therapy is given systemically, we observed no change in the frequency of Treg cells in the tumor, while the frequency actually increased in the draining lymph node (55). The route of administration may explain these differences as the same study showed that intratumoral administration of combined anti-OX40/anti-CTLA-4 did not affect Treg cells at a distant tumor site (56). However, Houot and Levy observed a reduction in Treg cells following systemic administration of combination therapy (63). These differences should be investigated further, but may be linked to the tumor model (lymphoma versus prostate-derived tumor), antibody clone (9D9 versus 9H10 for CTLA-4 blockade), dosing, or timing of therapy. In fact, the treatment regimen for combination immunotherapy, whether it is concurrent, staggered, or sequential, may prove very important in determining survival and immunological outcomes in cancer.

Another interesting observation from preclinical studies using combined anti-OX40/anti-CTLA-4 therapy was the induction of a population of Th2-cytokine secreting (IL-4^+^, IL-5^+^, IL-13^+^, IL-2^−^, TNF-α^−^) CD4 T cells (55). IL-4 was the primary driver of Th2 CD4 T cell differentiation as IL-4 blockade augmented the efficacy of combined anti-OX40/anti-CTLA-4 therapy in the TRAMP-C1 model. However, it remains unclear what immune subsets are affected by IL-4 or anti-IL-4 therapy. Furthermore, it is not known whether the expansion of Th2 CD4 T cells limits the efficacy of combination therapy. Previous work has demonstrated that low-affinity TCR signaling preferentially elicits Th2 CD4 T cell responses (100, 101). Whether this holds true following combined anti-OX40/anti-CTLA-4 therapy is currently an active area of investigation in our laboratory. If this hypothesis is correct, then one might speculate that a Th2 response could be redirected toward a more favorable Th1 response when combined with an antigen-specific vaccine, thereby boosting the efficacy of combination therapy without the need to block IL-4 in patients. Further studies to elucidate the connection between anti-OX40/anti-CTLA-4 combination therapy and IL-4 will be of interest as anti-OX40 progresses in the clinic.

### PD-1 blockade with OX40 agonism

A structural relative of CD28 and CD33, PD-1 is a transmembrane protein that plays a fundamental role in the inhibition of activated lymphocytes (102–106). High levels of PD-1 expression are frequently associated with populations of exhausted T cells, but robust expression of PD-1 has been observed in multiple subsets of activated lymphocytes, including T, B, and NK cells. PD-1 has two closely related ligands, PD-L1 and PD-L2, which are expressed by multiple cell types, and PD-L1 has a soluble isoform that can be secreted by some cancer cell lines and is detectable in the sera of some tumor-bearing hosts (107–109). PD-L1 is also abundantly expressed by numerous tumors and can be induced by exposure to both Type I and Type II interferons (110–113). Engagement of PD-1 on activated T cells decreases their capacity for a cytotoxic response following antigen recognition, suppresses proliferation, and potentiates apoptosis (107, 114–118). Both PD-1 and PD-L1 blockade have shown significant efficacy in a range of murine and human cancer models (103, 112, 119). Multiple studies have demonstrated that disruption of PD-1 signaling induced the expansion and cytolytic capacity of effector T cell populations and increased their infiltration into tumors (119–131). PD-1 blockade has also been shown to reduce the frequency and function of Treg cells within some tumors (124, 127, 128, 130, 132).

In clinical trials, checkpoint inhibition via PD-1 or PD-L1 blockade has shown substantial promise with respect to both efficacy and safety (133–136). In September 2014, The FDA approved the first anti-PD-1 mAb (pembrolizumab) for use in the treatment of advanced metastatic melanomas (137). Two additional anti-PD-1 mAbs, nivolumab and pidilizumab, and two anti-PD-L1 mAbs have also demonstrated encouraging efficacy in ongoing clinical trials (138–143). The unique mechanism of action responsible for the efficacy of PD-1/PD-L1 blockade makes this pathway an excellent candidate for combinatorial immunotherapies (58, 144). Indeed, combined blockade of PD-1 and CTLA-4 has generated impressive results in both preclinical and clinical trials (145). Disruption of PD-1 signaling can also synergize with LAG3 blockade, radiotherapy, BRAF inhibitors, and many other treatment strategies (146–150). However, one of the limitations of PD-1/PD-L1 blockade has been a difficulty in initiating a protective immune response against poorly immunogenic or large tumors (110, 112). Because of this, PD-1 blockade may be uniquely well suited for combinatorial immunotherapy strategies incorporating agents that support the development of tumor-reactive effector lymphocytes – treatments such as T and NK cell mAb agonists or tumor-specific immunization (119, 151, 152).

Administration of an OX40 agonist has multiple immunological effects that may complement the activity of PD-1/PD-L1 blockade. First, ligation of OX40 supports the expansion, survival, and effector function of activated CD4 and CD8 T cells, populations that express the PD-1 receptor. Second, OX40 co-stimulation has been reported to enhance the ability of T cells to respond productively to lower affinity antigens and OX40 ligation can enhance IFN-γ production by T cells in response to TCR stimulation. Because many cancerous cells up-regulate PD-L1 in response to IFN-γ exposure, PD-1/PD-L1 blockade may uniquely complement the therapeutic efficacy of OX40-driven effector lymphocytes within the tumor microenvironment. Furthermore, because cancerous cells may also up-regulate antigen presentation in response to IFN-γ exposure, the combination of PD-1 blockade and OX40 agonism may support a pro-inflammatory feedback loop within the tumor microenvironment that further augments anti-tumor immunity.

In a recent report, Guo and colleagues observed that PD-1 blockade synergized with an agonistic anti-OX40 mAb to promote regression of an implantable murine ovarian cancer, ID8, which was non-responsive to either monotherapy (59). The authors reported that the combination significantly increased the ratio of CD8 T cells at the tumor site (peritoneal cavity), relative to both Treg cells and myeloid-derived suppressor cells (MDSCs). They also reported that the combination of anti-OX40 and anti-PD-1 mAbs dramatically expanded peritoneal CD4 and CD8 effector memory cells, while reducing the frequency of the naïve T cells. They observed that tumor-resident T cells from the anti-PD-1/anti-OX40 group produced significantly higher levels of IFN-γ in response to PMA stimulation. In addition, splenocytes from these mice showed increased reactivity toward an ID8-specific antigen, mesothelin. Unsurprisingly, T cell depletion experiments indicated that the therapeutic effect was entirely dependent on the presence of CD8 T cells and partially dependent on CD4 T cells. Notably, the authors reported that cultured ID8 cancer cells expressed minimal PD-L1 or PD-L2, which may support the hypothesis that OX40-stimulated T cells induce PD-L1 expression at the tumor site via enhanced IFN-γ production. A separate study reported that triple combination therapy, using co-stimulatory anti-OX40 and anti-4-1BB mAbs and an inhibitory anti-PD-1 mAb, was uniquely effective in a murine hepatocellular carcinoma model, with enhanced tumor infiltration of cytotoxic effector T cells (153). Moving forward, a deeper understanding of the immunological interplay between PD-1/PD-L1 and OX40-targeted therapies will help identify and refine complementary therapeutic interventions. Although currently somewhat sparse, the existing body of evidence suggests PD-1 blockade is likely to synergize with OX40 agonists and may be particularly well suited for tumors that are naturally immunogenic and/or express high levels of PD-L1.

One issue that needs to be considered when moving these combinations to the clinic is the potential for increased toxicity and immune-related adverse events (irAEs). As described above, the results from a Phase 1 clinical trial (NCT01644968) conducted in patients with late-stage cancer indicated that OX40 immunotherapy was generally well tolerated. The majority of irAEs were relatively minor (Grade 1 and 2), while all of moderate to severe (Grade 3 and 4) irAEs were due to treatment-induced lymphopenia that was shown to be temporary (49). In comparison, a larger Phase 1 study of melanoma and renal cancer patients receiving anti-PD-1 observed moderate or severe irAEs in 14% (41/296) of patients (143). For anti-CTLA-4 monotherapy, the incidence of irAEs is highly dose-dependent but the frequency of moderate or severe irAEs for patients being treated with anti-CTLA-4 tends to be between 20 and 40% (154). Importantly, patients receiving a combination of anti-CTLA-4 and anti-PD-1 were more likely to experience Grade 3 or 4 irAEs (53%), although this combination yielded a substantially higher objective response rate than either monotherapy (155). The efficacy and toxicity of combining anti-OX40 therapy with either CTLA-4 or PD-1 has not been evaluated in humans, although Phase 1 clinical trials for both combinations are currently accruing patients (NCT02205333). Because anti-OX40 therapy augments the development, activity, and survival of effector lymphocyte populations, it is possible that the combination of anti-OX40 with checkpoint blockade will produce a higher frequency of irAEs than the respective individual treatments. However, synergy between anti-OX40 therapy and checkpoint blockade may yield objective responses at lower dosages than are required when each drug is used as a monotherapy. Overall, the ongoing clinical experience with anti-CTLA-4 suggests that modulating dosages and clinically managing irAEs can be an effective strategy to alleviate symptoms and maintain patients on treatment (156).

### On the horizon: Other checkpoint inhibitors with potential synergy

Like OX40, CTLA-4, and PD-1, T cells express numerous cell surface receptors capable of modulating an anti-tumor immune response. Here, we touch on several surface receptors that when targeted may synergize with OX40 agonists (Figure 1). A member of the immunoglobulin super-family, Lymphocyte Activation Gene 3 (LAG3) is expressed by subsets of both activated and exhausted lymphocytes and plasmacytoid dendritic cells, and plays a key role in regulating the effector activity of tumor-associated lymphocytes (145, 157, 158). In some tumors, LAG3 is highly expressed by infiltrating effector T cells. LAG3 is known to interact with MHC-II receptors, and blockade of LAG3 has been shown to support effector T cell activity both *in vitro* and *in vivo* (159–164). Experiments suggest a synergistic interaction between combination anti-PD-1 and anti-LAG3 therapies that appears to enhance anti-tumor immunity, in part by preventing exhaustion and anergy in effector T cell populations (146, 149, 165). Although no studies have yet been published that directly evaluate the efficacy of combined anti-LAG3/anti-OX40 treatment, the current understanding of the mechanisms that underlie each therapy suggest the potential for cooperative activity. Specifically, OX40 agonists induce expansion and infiltration of effector T cells into the tumor, and the cytotoxic activity of these cells in the tumor microenvironment may be supported by LAG3 blockade. Additionally, many tumor cells and APCs up-regulate MHC-II expression in response to IFN-γ exposure and because OX40 therapy increases IFN-γ production by infiltrating T cells, there may be a rational basis for evaluating this combination.

Another possible target for combination therapy with anti-OX40 mAb is through targeted blockade of killer immunoglobulin-like receptors (KIRs). Primarily expressed by NK cells, KIRs are a class of transmembrane proteins that are important regulators of antigen-specificity and cytotoxic activity (166, 167). KIR family receptors are known to interact with MHC molecules on adjacent cells, with each KIR having specificity for different MHC subsets (168). KIRs can transduce either activating or inhibitory signals, and the balance between these signals is critical for mediating both self-tolerance and cytolytic activity. As a cancer immunotherapy, blockade of inhibitory KIRs using mAbs has demonstrated promise in murine tumor models and is being evaluated in early-stage clinical trials (169–172). Although the direct combination of OX40 agonism and inhibitory KIR blockade has not been reported, one might surmise that this combination might induce a potent anti-tumor response. Inhibitory KIR blockade, which can induce NK-cell-mediated tumor lysis, may promote the release of tumor-associated antigens. These antigens may provide TCR stimulation to OX40-stimulated T cells, thereby enhancing the effects of anti-OX40 therapy. It will be interesting to see what clinical trials unfold, and whether these combinations will be tested in the near future.

T cell immunoglobulin mucin 3 (TIM3) and B- and T-lymphocyte attenuator (BTLA, CD272) are also cell surface receptors that are expressed by effector T cell populations and transduce inhibitory signals (145). TIM3 is expressed on tumor-reactive CD8 T cells and antibody-mediated blockade of TIM3 enhanced their ability to produce IFN-γ (173). Galectin-9, which is highly expressed by some tumors, has been reported to be the natural ligand for TIM3, although this relationship is somewhat controversial (174, 175). Blockade of TIM3 promoted tumor regression, both as a monotherapy and in combination with anti-PD-1 (173, 176, 177). BTLA is expressed at a high level by tumor-reactive CD8 T cells and interaction with its ligand, HVEM, can inhibit the functional activity of this population (178, 179). Because HVEM is expressed by some tumors, and BTLA-deficient mice mount more robust T cell responses, blockade of this target has been proposed as a cancer immunotherapy (180–182). Similar to LAG3 blockade, targeting TIM3 and/or BTLA may augment the efficacy of OX40 therapy by supporting the expansion, survival, and cytotoxic effector function of lymphocytes, particularly within the microenvironment of the tumor (183–186).

Beyond checkpoint inhibition, several other therapeutic interventions have been reported to complement the anti-tumor activity of OX40 stimulation, including other immune-stimulatory mAbs, recombinant IL-2, immunization, radiotherapy, intratumoral TLR ligands, chemotherapeutics, and more (26, 53, 54, 63, 65, 70, 71, 80, 153, 187–192). One study investigated the combination of anti-OX40 mAbs and the chemotherapeutic cyclophosphamide, which is known to activate tumor-reactive T cells and selectively deplete Treg cells. This combination initiated tumor regression in the poorly immunogenic B16 melanoma model and induced a potent anti-tumor T cell response (65). Mechanistic studies revealed that anti-OX40 and cyclophosphamide induced a memory CD4 T cell population capable of producing both Th1 and Th2 cytokines (66). Other investigators have looked at the use of anti-OX40 surgical resection or radiation of the tumor. These experiments demonstrated that an OX40 agonist administered at the time of resection prevented local disease recurrence, and when combined with radiation prolonged survival and the frequency of disease-free animals (26). These studies contributed to the design of two clinical trials; one examining radiation, cyclophosphamide, and anti-OX40 in patients with metastatic prostate cancer (NCT01303705); and the other using combination stereotactic body radiation and anti-OX40 in patients with metastatic breast cancer (NCT01862900).

### Triple combinations and beyond…

Because anti-tumor immunity is directed by a dynamic constellation of signals, maximizing the therapeutic benefit of lymphocyte agonists, such as anti-OX40 mAbs, will likely depend on incorporating multiple complementary interventions. The most viable candidates for combinatorial therapies are those that have already achieved FDA-approval, especially since anti-OX40 itself remains experimental. One particularly intriguing possibility is a triple combination of OX40 agonism with concomitant PD-1 and CTLA-4 blockade. The distinct mechanisms underlying PD-1 and CTLA-4 blockade have already been shown to synergize in the treatment of many murine and human cancers (135, 140, 150, 155, 193, 194). A “triple threat” immunotherapy approach that includes OX40 stimulation may help augment the efficacy of dual PD-1/CTLA-4 blockade by enhancing the expansion, survival, and cytolytic activity of tumor-reactive effector T cells. Other FDA-approved therapies, such as recombinant IL-2 and radiotherapy, may also be well suited for multiplex immunotherapy approaches that incorporate both OX40 stimulation and checkpoint inhibitor blockade. Administration of IL-2 can augment the activity of OX40 stimulation, in part because it stimulates the proliferation of T cells and their up-regulation of the OX40 receptor through a JAK3/STAT5-dependent mechanism (71, 195–199). Radiotherapy can also complement OX40 treatment and may be a valuable tool for disrupting the immune suppressive microenvironment of established tumors and releasing tumor-associated antigens for recognition by the immune system (26, 80, 147, 153, 200, 201). Because each of the aforementioned approaches utilizes a distinct mechanism, varied combinations of these strategies may yield unique therapeutic efficacy when combined with OX40 agonism (Table 1). Indeed, overcoming the challenge of mounting a curative immune response in a diverse population of patients will almost certainly require multiple complementary therapeutic modalities to overcome the immunosuppressive tumor microenvironment of established tumors and provide a protective anti-tumor immune response.

## Author Contributions

SL and MM reviewed the relevant literature and drafted the manuscript. SL, MM, and WR revised the manuscript. All authors read and approved the final manuscript.

## Conflict of Interest Statement

William L. Redmond receives research support from Bristol-Myers Squibb and Galectin Therapeutics. The other co-authors declare that the research was conducted in the absence of any commercial or financial relationships that could be construed as a potential conflict of interest.
